# Development and validation of a prediction model for 1-year all-cause rehospitalisation after discharge in patients with heart failure with preserved ejection fraction based on the systemic immune-inflammation index

**DOI:** 10.3389/fcvm.2026.1821564

**Published:** 2026-05-08

**Authors:** Pan Chen, Xiaoyan Yin, Boshi Liu, Lei Ren

**Affiliations:** 1 Department of Cardiovascular Medicine, Anhui Medical University Affiliated Fuyang People's Hospital, Fuyang People's Hospital, Fuyang, Anhui, China; 2 Department of Cardiovascular Medicine, Bengbu Medical University Affiliated Fuyang Hospital, Fuyang People's Hospital, Fuyang, Anhui, China

**Keywords:** all-cause rehospitalisation, heart failure with preserved ejection fraction, systemic immune-inflammation index, nomogram, prognosis

## Abstract

**Objective:**

To develop and internally validate a practical model for predicting the risk of at least one all-cause rehospitalisation within 1 year after discharge in patients with heart failure with preserved ejection fraction (HFpEF), with particular attention to the systemic immune-inflammation index (SII).

**Methods:**

This single-center observational study was based on retrospective analysis of index-admission data and follow-up records from patients with HFpEF treated at Fuyang People's Hospital between January 2022 and January 2024. Among 341 initially eligible patients, 29 were lost to follow-up, leaving a final analytic cohort of 312 patients. The cohort was divided into a training set (*n* = 218) and a validation set (*n* = 94) using outcome-stratified random sampling. The primary endpoint was the occurrence of at least one all-cause rehospitalisation within 1 year after discharge. Candidate predictors were screened by least absolute shrinkage and selection operator logistic regression in the training set, followed by multivariable logistic regression to construct the final model and nomogram. Model performance was assessed by discrimination, calibration, and decision curve analysis.

**Results:**

LnSII, LnNT-proBNP, LnLp(a), GFR, and hypertension remained in the final model. The model showed good discrimination, with an area under the receiver operating characteristic curve of 0.8468 in the training set and 0.8302 in the validation set. At the optimal cutoff derived from the training set, sensitivity and specificity were 0.7101 and 0.8993 in the training set and 0.5333 and 0.8750 in the validation set, respectively. Calibration was acceptable in both cohorts according to the Hosmer–Lemeshow test, and decision curve analysis suggested potential clinical utility.

**Conclusion:**

A 5-variable model incorporating LnSII, LnNT-proBNP, LnLp(a), GFR, and hypertension showed good discrimination, acceptable calibration, and potential clinical usefulness for predicting 1-year all-cause rehospitalisation after discharge in patients with HFpEF. External validation is needed before broader application.

## 1 Introduction

HFpEF is common, heterogeneous, and often followed by repeat hospitalizations after discharge. Although recent trials have improved the therapeutic landscape of HFpEF, recurrent hospitalisation after discharge remains common in routine practice (1–4). Existing prognostic models for HFpEF also vary in predictor selection, performance, and clinical applicability, which limits their value in everyday risk stratification (5).

Systemic inflammation is increasingly recognised as an important component of HFpEF. Inflammatory activation, endothelial dysfunction, vascular injury, and cardiorenal interaction may jointly contribute to congestion, impaired reserve, and subsequent clinical instability (6, 7). The systemic immune-inflammation index (SII), calculated from platelet, neutrophil, and lymphocyte counts, is an inexpensive marker that reflects both inflammatory burden and thrombo-inflammatory imbalance. Recent studies have shown that elevated SII is associated with adverse outcomes in patients with HFpEF (8, 9). In addition to inflammatory status, NT-proBNP remains a well-established indicator of haemodynamic stress, impaired renal function is closely linked to worse prognosis, and lipoprotein(a) [Lp(a)] may provide complementary information on vascular and inflammatory risk (7, 10).

This study aimed to develop and internally validate a prediction model for the risk of at least one all-cause rehospitalisation within 1 year after discharge in patients with HFpEF using routinely available clinical, laboratory, and echocardiographic variables, with particular attention to the potential contribution of SII. A nomogram was further constructed to facilitate individualised risk estimation in clinical practice.

## 2 Materials and methods

### 2.1 General information

This was a single-center observational study based on retrospective analysis of index-admission data and follow-up records from patients with heart failure with preserved ejection fraction (HFpEF) treated at Fuyang People's Hospital between January 2022 and January 2024. A total of 341 consecutive patients met the initial screening criteria and were scheduled for 1-year post-discharge follow-up through review of electronic medical records, outpatient records, and telephone contact. Twenty-nine patients were lost to follow-up, leaving a final analytic cohort of 312 patients. After exclusion of patients lost to follow-up, the final cohort was randomly divided in a 7:3 ratio into a training cohort (*n* = 218) and a validation cohort (*n* = 94). Outcome-stratified random sampling based on rehospitalisation status was used to preserve a similar event proportion in the training and validation cohorts.

Inclusion criteria were as follows: (1) age ≥18 years; (2) diagnosis of HFpEF according to the 2022 AHA/ACC/HFSA guideline, defined by symptoms and/or signs of heart failure, left ventricular ejection fraction (LVEF) ≥50%, and objective evidence supporting elevated filling pressure, including elevated natriuretic peptides and/or structural or functional cardiac abnormalities consistent with diastolic dysfunction; (3) availability of baseline demographic, clinical, laboratory, and echocardiographic data from the index admission; and (4) availability of 1-year follow-up information.

Exclusion criteria were as follows: (1) severe valvular heart disease (moderate or greater stenosis or regurgitation), constrictive pericarditis, hypertrophic cardiomyopathy, infiltrative cardiomyopathy, or acute coronary syndrome; (2) severe hepatic impairment (Child–Pugh class C) or severe renal impairment (estimated glomerular filtration rate < 15 mL/min/1.73 m^2^); (3) active malignancy or terminal illness with life expectancy <1 year; (4) pregnancy; and (5) factors likely to compromise follow-up quality or data integrity.

### 2.2 Study design and model development

The primary endpoint was a binary outcome defined as the occurrence of at least one all-cause rehospitalisation within 1 year after discharge from the index admission. A total of 45 pre-specified baseline variables were considered as candidate predictors, including demographic characteristics, comorbidities, laboratory indices, and echocardiographic parameters. All 45 candidate variables were entered into the LASSO model without prior clinical reduction. Variable selection was performed in the training cohort using least absolute shrinkage and selection operator (LASSO) logistic regression with 10-fold cross-validation. The *λ*1se criterion, defined as the largest *λ* within 1 standard error of the minimum cross-validation error, was selected to obtain a more parsimonious model. Variables retained by LASSO were then entered into multivariable logistic regression to establish the final model. A nomogram was subsequently developed on the basis of the final multivariable model. Model performance was evaluated in the training and validation cohorts by discrimination, calibration, and clinical utility, including receiver operating characteristic (ROC) analysis, sensitivity, specificity, the Hosmer–Lemeshow test, calibration curves, and decision curve analysis (DCA).

### 2.3 Observation indicators

Baseline variables were collected from the index admission. Demographic and clinical variables included age, sex, height, weight, body mass index (BMI), systolic blood pressure (SBP), diastolic blood pressure (DBP), heart rate, smoking status, drinking status, hypertension, type 2 diabetes mellitus (T2DM), coronary heart disease (CHD), transient ischaemic attack (TIA), chronic obstructive pulmonary disease (COPD), and New York Heart Association (NYHA) functional class.

Laboratory variables included white blood cell count (WBC), neutrophil count (N), lymphocyte count (LYM), haemoglobin (Hb), platelet count (Plt), C-reactive protein (CRP), alanine aminotransferase (ALT), aspartate aminotransferase (AST), albumin (ALB), thyroid function indices, creatinine (Cr), uric acid (UA), triglycerides (TG), total cholesterol (TC), high-density lipoprotein cholesterol (HDL), low-density lipoprotein cholesterol (LDL), lipoprotein(a) [Lp(a)], and N-terminal pro-B-type natriuretic peptide (NT-proBNP).

Echocardiographic variables included left ventricular ejection fraction (LVEF), left atrial diameter (LA), interventricular septal thickness (IVS), and left ventricular posterior wall thickness (LVPW). Estimated glomerular filtration rate (GFR) was obtained from the laboratory report of the index admission and was calculated from serum creatinine using the standard equation adopted by the hospital laboratory system.

The systemic immune-inflammation index (SII) was calculated as platelet count × neutrophil count/lymphocyte count. Because SII, NT-proBNP, and Lp(a) showed right-skewed distributions, natural logarithmic transformation was performed before model development, generating LnSII, LnNT-proBNP, and LnLp(a).

### 2.4 Statistical treatment

Statistical analyses were performed using SPSS 27.0 and R 4.5.2.Patients without outcome assessment were excluded from the final analysis. For baseline numeric variables with missing values, median imputation was performed before model development. No multiple imputation was performed. Continuous variables with approximately normal distributions are presented as mean ± standard deviation, whereas non-normally distributed variables are presented as median (interquartile range). Categorical variables are presented as number (percentage). Group comparisons were performed using the independent-samples *t* test or Mann–Whitney U test for continuous variables and the chi-square test or Fisher's exact test for categorical variables, as appropriate. Spearman correlation analysis and variance inflation factor analysis were performed before multivariable modelling to assess collinearity among the final predictors. All tests were two-sided, and *P* < 0.05 was considered statistically significant.

## 3 Results

### 3.1 Correlation analysis and multicollinearity assessment

Before model development, collinearity among the final predictors was assessed. Spearman correlation analysis showed no strong pairwise correlation. The largest absolute correlation coefficient was observed between LnSII and LnNT-proBNP (*r* = −0.477), whereas the absolute correlation coefficients among the remaining predictors were all ≤0.124. Variance inflation factor analysis further showed that LnSII, LnNT-proBNP, LnLp(a), GFR, and hypertension had VIF values of 1.328, 1.319, 1.019, 1.041, and 1.018, respectively, indicating no evident multicollinearity.

### 3.2 Baseline comparison between patients with and without 1-year rehospitalisation

A total of 312 patients were included in the final analysis. Among them, 99 patients (31.73%) experienced at least one all-cause rehospitalisation within 1 year after discharge, whereas 213 patients (68.27%) did not. Significant between-group differences were observed for LnNT-proBNP, LnSII, platelet count, GFR, HDL, NT-proBNP, CRP, lymphocyte count, SII, Lp(a), LnLp(a), hypertension, TIA, and NYHA class (Table 1).

**Table 1:** Baseline characteristics of patients with and without 1-year all-cause rehospitalisation.

Variables	Total (*n* = 312)	No rehospitalisation (*n* = 213)	Rehospitalisation (*n* = 99)	Statistic	*P*
Height, Mean ± SD	1.65 ± 0.07	1.66 ± 0.07	1.65 ± 0.07	*t* = 1.40	0.162
Weight, Mean ± SD	68.33 ± 8.34	68.44 ± 8.11	68.08 ± 8.86	*t* = 0.36	0.722
BMI, Mean ± SD	25.11 ± 3.75	25.04 ± 3.65	25.26 ± 3.96	*t* = −0.48	0.631
SBP, Mean ± SD	145.71 ± 18.79	146.30 ± 18.24	144.42 ± 19.96	*t* = 0.82	0.413
DBP, Mean ± SD	80.75 ± 12.39	80.99 ± 12.74	80.24 ± 11.63	*t* = 0.49	0.622
LnNT-proBNP, Mean ± SD	8.15 ± 0.62	8.07 ± 0.60	8.34 ± 0.61	*t* = −3.63	**<** **.** **001**
WBC, Mean ± SD	6.21 ± 1.14	6.21 ± 1.08	6.21 ± 1.26	*t* = 0.03	0.975
N, Mean ± SD	3.90 ± 1.05	3.85 ± 1.01	4.02 ± 1.11	*t* = −1.31	0.192
LnSII, Mean ± SD	6.35 ± 0.52	6.27 ± 0.50	6.54 ± 0.50	*t* = −4.50	**<** **.** **001**
Hb, Mean ± SD	125.13 ± 18.56	125.21 ± 18.94	124.96 ± 17.80	*t* = 0.11	0.911
Plt, Mean ± SD	220.07 ± 57.02	213.16 ± 57.43	234.95 ± 53.43	*t* = −3.19	**0** **.** **002**
FT3, Mean ± SD	3.12 ± 0.44	3.12 ± 0.44	3.12 ± 0.44	*t* = −0.08	0.934
FT4, Mean ± SD	1.04 ± 0.15	1.03 ± 0.15	1.07 ± 0.15	*t* = −1.93	0.054
ALT, Mean ± SD	24.57 ± 9.85	23.83 ± 9.48	26.15 ± 10.47	*t* = −1.94	0.053
AST, Mean ± SD	23.34 ± 9.01	23.22 ± 8.88	23.59 ± 9.31	*t* = −0.33	0.741
ALB, Mean ± SD	40.78 ± 4.27	41.02 ± 4.01	40.27 ± 4.74	*t* = 1.43	0.152
GFR, Mean ± SD	113.22 ± 19.20	116.03 ± 19.13	107.16 ± 18.01	*t* = 3.88	**<** **.** **001**
TC, Mean ± SD	4.84 ± 0.89	4.78 ± 0.94	4.96 ± 0.79	*t* = −1.69	0.093
HDL, M (Q₁, Q₃)	1.10 (0.90, 1.30)	1.10 (1.00, 1.30)	1.10 (0.90, 1.20)	*Z* = −2.38	**0** **.** **017**
Age, M (Q₁, Q₃)	70.00 (63.75, 73.00)	70.00 (63.00, 72.00)	71.00 (65.50, 73.00)	*Z* = −1.07	0.283
LVEF, M (Q₁, Q₃)	56.00 (52.00, 61.00)	55.00 (52.00, 61.00)	57.00 (52.00, 61.50)	*Z* = −0.89	0.373
LA, M (Q₁, Q₃)	36.00 (33.00, 39.00)	36.00 (33.00, 39.00)	36.00 (33.00, 39.00)	*Z* = −0.14	0.893
LVPW, M (Q₁, Q₃)	10.00 (9.00, 11.00)	10.00 (9.00, 11.00)	10.00 (9.00, 11.00)	*Z* = −0.19	0.851
IVS, M (Q₁, Q₃)	11.00 (10.00, 12.00)	11.00 (10.00, 12.00)	11.00 (10.00, 12.00)	*Z* = −1.17	0.242
Smoke, M (Q₁, Q₃)	0.00 (0.00, 18.00)	3.00 (0.00, 19.00)	0.00 (0.00, 16.00)	*Z* = −1.56	0.119
Drink, M (Q₁, Q₃)	0.00 (0.00, 15.00)	1.00 (0.00, 15.00)	0.00 (0.00, 12.00)	*Z* = −1.45	0.148
Pulse, M (Q₁, Q₃)	78.00 (72.00, 84.00)	79.00 (72.00, 84.00)	77.00 (71.00, 83.00)	*Z* = −1.02	0.309
NT-proBNP, M (Q₁, Q₃)	3623.00 (2360.75, 5261.75)	3349.00 (2289.00, 4618.00)	4187.00 (2952.00, 6140.50)	*Z* = −3.48	**<** **.** **001**
CRP, M (Q₁, Q₃)	4.00 (2.50, 5.40)	3.40 (2.00, 5.20)	4.90 (3.45, 6.60)	*Z* = −5.25	**<** **.** **001**
LYM, M (Q₁, Q₃)	1.48 (1.20, 1.76)	1.54 (1.26, 1.78)	1.33 (1.06, 1.62)	*Z* = −3.36	**<** **.** **001**
SII, M (Q₁, Q₃)	566.25 (405.38, 816.98)	519.70 (385.40, 749.10)	645.80 (509.95, 964.45)	*Z* = −4.26	**<** **.** **001**
TSH, M (Q₁, Q₃)	1.87 (1.25, 2.34)	1.82 (1.22, 2.33)	1.89 (1.33, 2.34)	*Z* = −0.85	0.397
Cr, M (Q₁, Q₃)	95.80 (78.92, 112.63)	97.80 (81.30, 113.60)	93.50 (75.75, 111.30)	*Z* = −0.92	0.356
UA, M (Q₁, Q₃)	363.05 (304.82, 415.27)	366.90 (306.60, 417.80)	349.10 (293.75, 409.70)	*Z* = −0.99	0.321
TG, M (Q₁, Q₃)	2.00 (1.20, 2.70)	1.90 (1.20, 2.70)	2.10 (1.30, 2.80)	*Z* = −0.58	0.564
Lp(a), M (Q₁, Q₃)	328.35 (271.62, 380.90)	307.30 (258.20, 364.60)	355.50 (308.65, 426.00)	*Z* = −4.66	**<** **.** **001**
LnLp(a), M (Q₁, Q₃)	5.79 (5.60, 5.94)	5.73 (5.55, 5.90)	5.87 (5.73, 6.05)	*Z* = −4.66	**<** **.** **001**
Gender, n(%)				*χ*^2^ = 3.57	0.059
Male	160 (51.28)	117 (54.93)	43 (43.43)		
Female	152 (48.72)	96 (45.07)	56 (56.57)		
Hypertension, n(%)				*χ*^2^ = 10.66	**0** **.** **001**
No	207 (66.35)	154 (72.30)	53 (53.54)		
Yes	105 (33.65)	59 (27.70)	46 (46.46)		
T2DM, n(%)				*χ*^2^ = 1.66	0.198
No	267 (85.58)	186 (87.32)	81 (81.82)		
Yes	45 (14.42)	27 (12.68)	18 (18.18)		
CHD, n(%)				*χ*^2^ = 3.23	0.072
No	271 (86.86)	190 (89.20)	81 (81.82)		
Yes	41 (13.14)	23 (10.80)	18 (18.18)		
TIA, n(%)				*χ*^2^ = 17.63	**<** **.** **001**
No	277 (88.78)	200 (93.90)	77 (77.78)		
Yes	35 (11.22)	13 (6.10)	22 (22.22)		
COPD, n(%)				*χ*^2^ = 3.56	0.059
No	277 (88.78)	194 (91.08)	83 (83.84)		
Yes	35 (11.22)	19 (8.92)	16 (16.16)		
NYHA, n(%)				*χ*^2^ = 8.38	**0** **.** **015**
I	80 (25.64)	65 (30.52)	15 (15.15)		
II	120 (38.46)	77 (36.15)	43 (43.43)		
III	112 (35.90)	71 (33.33)	41 (41.41)		

*t* denotes the *t*-test statistic, *Z* denotes the Mann–Whitney U test statistic, and *χ*^2^ denotes the chi-square statistic; Q1 and Q3 indicate the first and third quartiles. HFpEF, heart failure with preserved ejection fraction; BMI, body mass index; SBP, systolic blood pressure; DBP, diastolic blood pressure; LVEF, left ventricular ejection fraction; LVPW, left ventricular posterior wall thickness; IVS, interventricular septal thickness; T2DM, type 2 diabetes mellitus; CHD, coronary heart disease; TIA, transient ischaemic attack; COPD, chronic obstructive pulmonary disease; NYHA, New York Heart Association functional class; GFR, glomerular filtration rate; NT-proBNP, N-terminal pro-B-type natriuretic peptide; Lp(a), lipoprotein(a); SII, systemic immune-inflammation index.

Bold values indicate statistical significance at *P* < 0.05.

### 3.3 Feature selection

**Figure 1:**
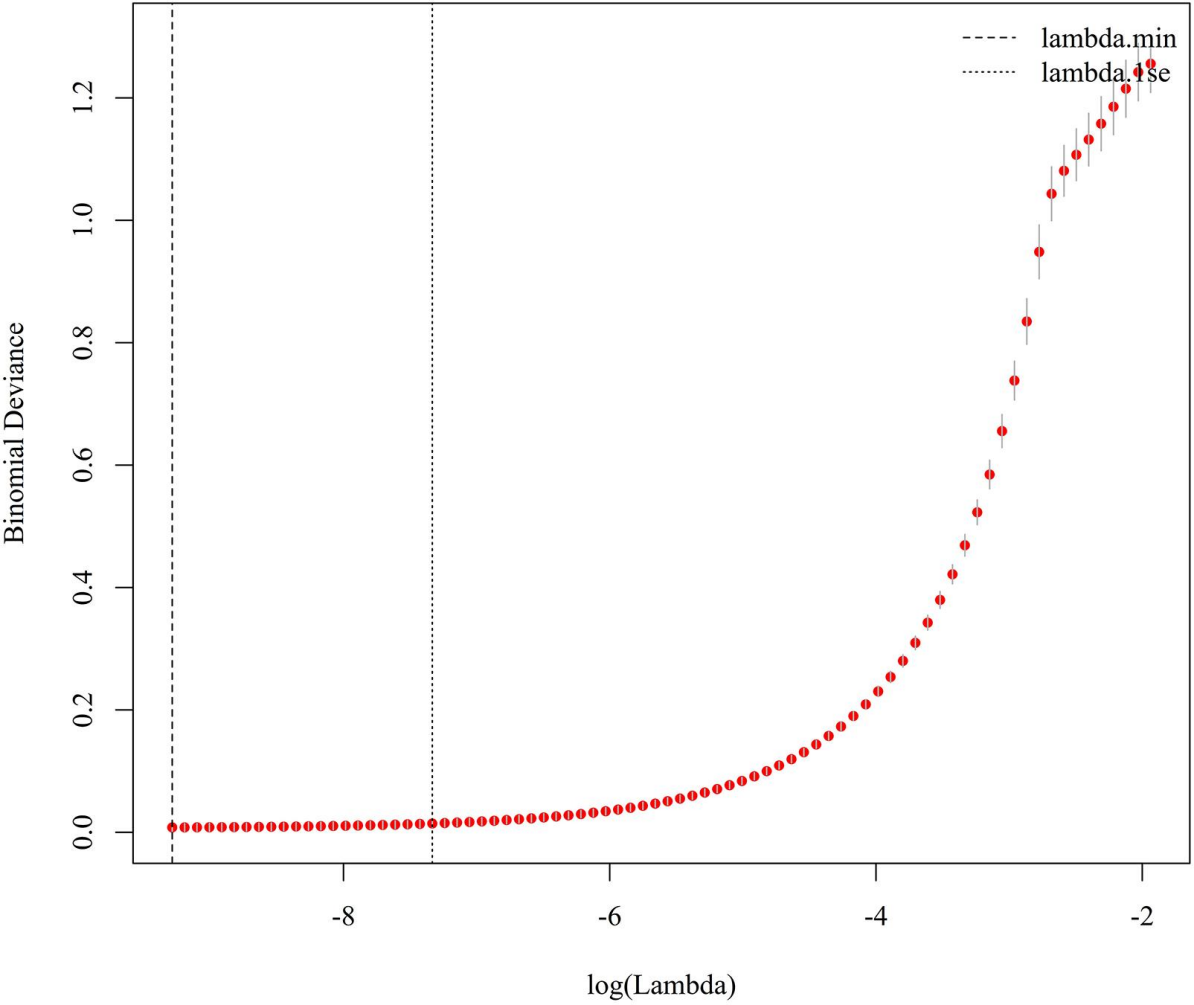
Ten-fold cross-validation curve for LASSO logistic regression. The binomial deviance is plotted against log(lambda), and the vertical lines indicate lambda.min and lambda.1se.

**Figure 2:**
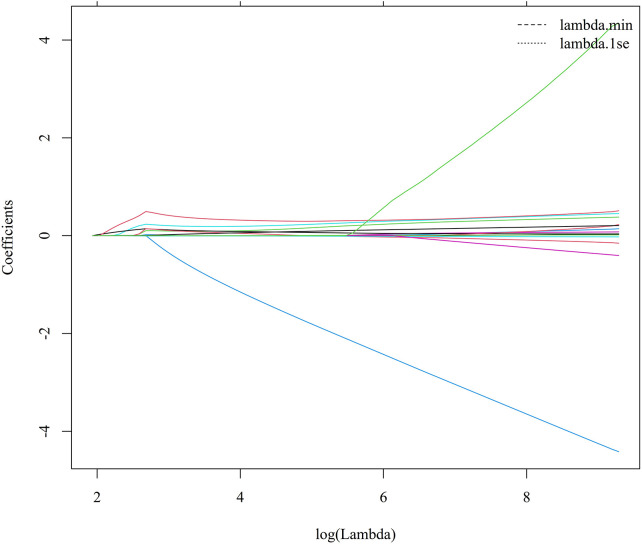
LASSO coefficient profiles of candidate predictors. Each curve represents the coefficient trajectory of one predictor across different values of log(lambda).

LASSO logistic regression with 10-fold cross-validation was performed in the training cohort. The minimum cross-validation error was observed at *λ*_min = 9.27 × 10^−5, and *λ*_1se was 6.54  ×  10^−4. At *λ*_1se, 15 variables with non-zero coefficients were retained, namely LVEF, IVS, hypertension, CHD, TIA, NYHA class, CRP, LnSII, ALT, ALB, GFR, LDL, Lp(a), LnNT-proBNP, and LnLp(a) (Figures 1, 2).

### 3.4 Multivariable logistic regression model

Variables retained by LASSO were further entered into multivariable logistic regression in the training cohort. As shown in Table 2, LnSII, LnNT-proBNP, LnLp(a), GFR, and hypertension remained independently associated with the risk of 1-year all-cause rehospitalisation. Table 2 presents *β* coefficients with standard errors, together with the corresponding odds ratios. For LnSII, LnNT-proBNP, and LnLp(a), the reported odds ratios correspond to a 1-unit increase on the natural log scale; for GFR, the odds ratio corresponds to a 1 mL/min/1.73 m^2^ increase; hypertension was analysed as yes versus no.

**Table 2:** Multivariable logistic regression results for 1-year all-cause rehospitalisation.

Variable	*β* (SE)	OR (95% CI)	*P* value
LnSII (per 1-unit increase)	1.89 (0.48)	6.65 (2.62–16.89)	<0.001
LnNT-proBNP (per 1-unit increase)	1.96 (0.37)	7.09 (3.45–14.56)	<0.001
LnLp(a) (per 1-unit increase)	3.02 (0.74)	20.55 (4.82–87.59)	<0.001
GFR (per 1 mL/min/1.73 m^2^ increase)	−0.03 (0.01)	0.97 (0.95–0.99)	0.004
Hypertension (yes vs. no)	1.02 (0.39)	2.78 (1.29–5.96)	0.009

*β* denotes the regression coefficient and SE denotes the standard error. LnSII, natural logarithm of the systemic immune-inflammation index; LnNT-proBNP, natural logarithm of N-terminal pro-B-type natriuretic peptide; LnLp(a), natural logarithm of lipoprotein(a); GFR, glomerular filtration rate.

### 3.5 Nomogram construction

**Figure 3:**
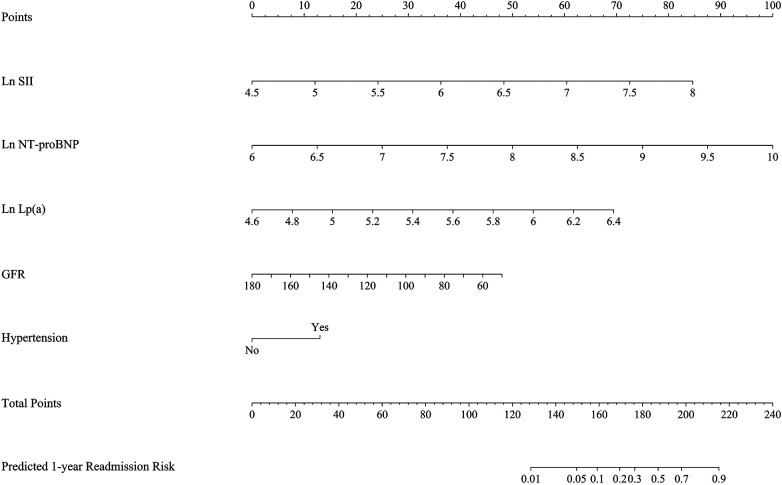
Nomogram for predicting 1-year all-cause rehospitalisation after discharge in patients with HFpEF. The nomogram incorporates LnSII, LnNT-proBNP, LnLp(a), GFR, and hypertension status.

A nomogram was constructed on the basis of the final multivariable model, incorporating LnSII, LnNT-proBNP, LnLp(a), GFR, and hypertension status. The total score derived from the nomogram was used to estimate the individual probability of at least one all-cause rehospitalisation within 1 year after discharge (Figure 3).

### 3.6 Evaluation of model performance

**Figure 4:**
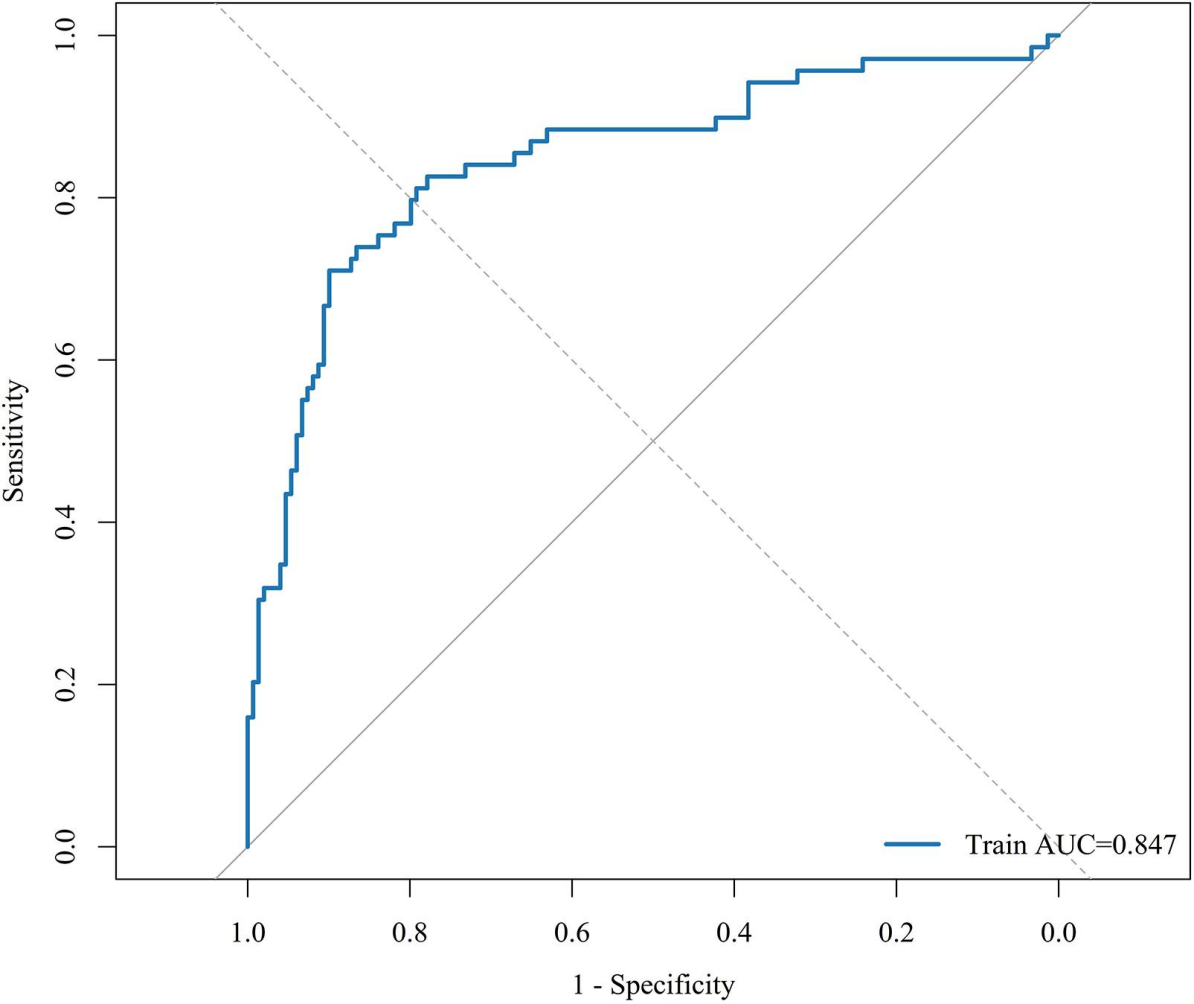
Receiver operating characteristic curve of the final model in the training cohort. The area under the curve was 0.8468.

**Figure 5:**
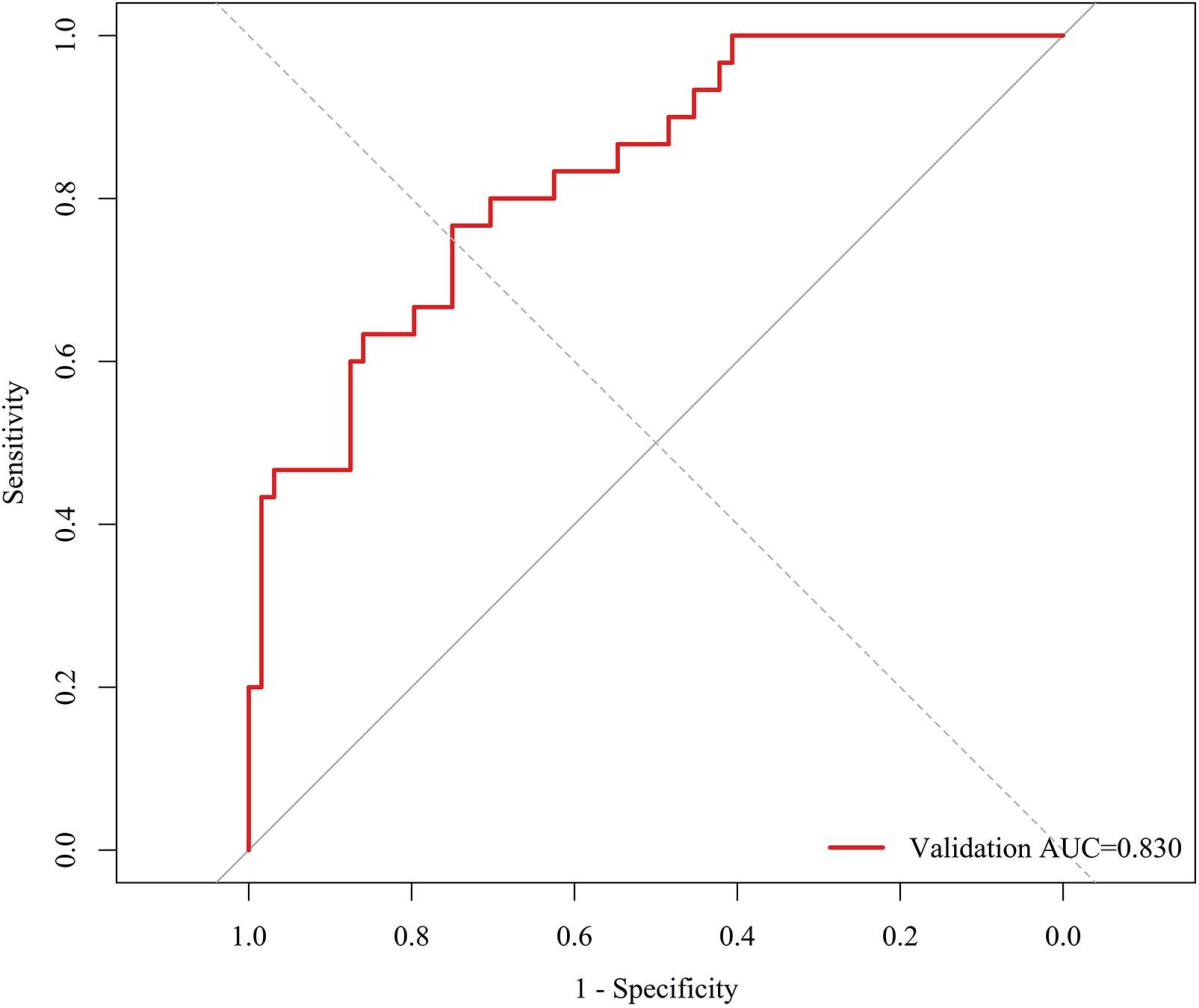
Receiver operating characteristic curve of the final model in the validation cohort. The area under the curve was 0.8302.

The final model was developed in the training cohort (*n* = 218; 69 rehospitalisation events, 31.65%) and tested in the validation cohort (*n* = 94; 30 rehospitalisation events, 31.91%). ROC analysis showed good discrimination, with an AUC of 0.8468 in the training cohort and 0.8302 in the validation cohort (Figures 4, 5). Using the optimal cutoff value derived from the training ROC curve (0.451042), the sensitivity and specificity were 0.7101 and 0.8993, respectively, in the training cohort, and 0.5333 and 0.8750, respectively, in the validation cohort.

### 3.7 Calibration assessment

**Figure 6:**
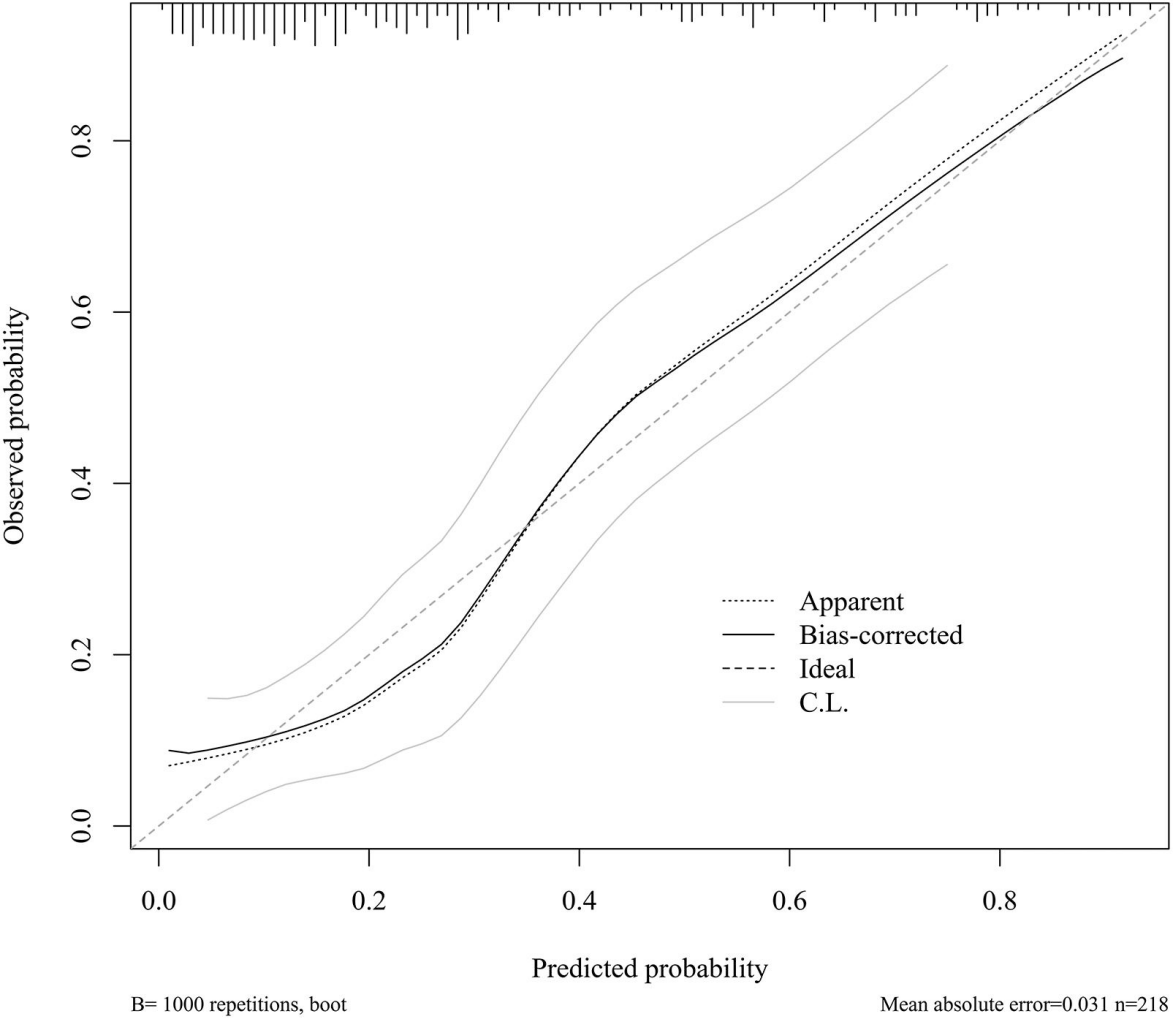
Calibration curve of the final model for predicting 1-year all-cause rehospitalisation. The apparent, bias-corrected, ideal, and confidence-limit curves are shown.

Calibration was assessed using the Hosmer–Lemeshow goodness-of-fit test and calibration curves. In the training cohort, the Hosmer–Lemeshow test showed acceptable agreement between predicted and observed risk (*χ*^2^ = 12.89, *P* = 0.1157). In the validation cohort, calibration was also acceptable (*χ*^2^ = 3.99, *P* = 0.8580). The calibration plots further suggested reasonable agreement between predicted and observed probabilities (Figure 6).

### 3.8 Clinical utility

**Figure 7:**
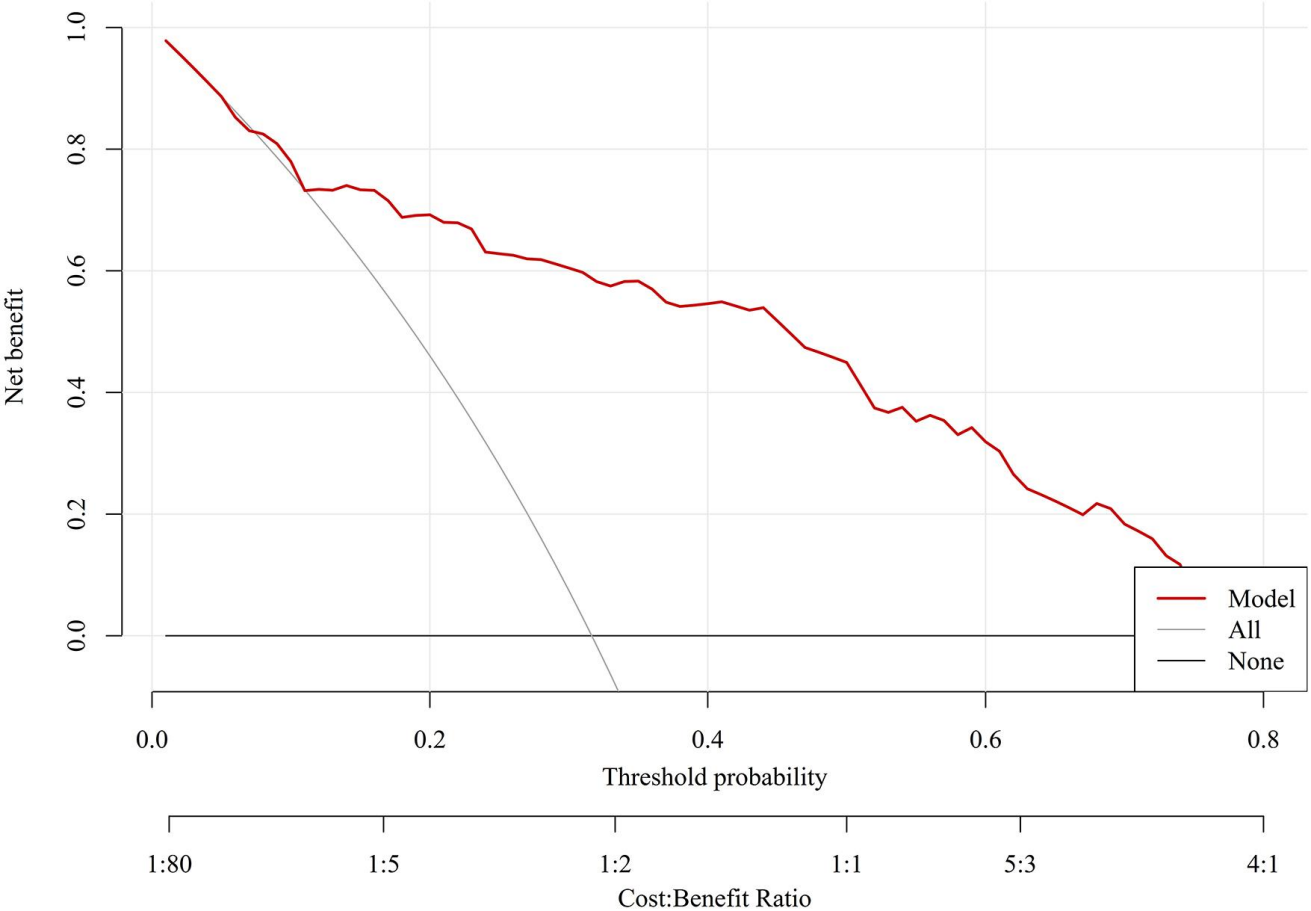
Decision curve analysis of the final model. The net benefit of the model is compared with the treat-all and treat-none strategies across threshold probabilities.

Decision curve analysis showed that the model provided positive net clinical benefit across a broad range of threshold probabilities in both the training and validation cohorts, supporting its potential clinical usefulness (Figure 7).

## 4 Discussion

In this single-center HFpEF cohort, a model using LnSII, LnNT-proBNP, LnLp(a), GFR, and hypertension predicted 1-year all-cause rehospitalisation with reasonable accuracy. Discrimination was good in both cohorts, calibration was acceptable, and decision curve analysis suggested potential clinical usefulness. All five predictors are routinely available before discharge, so the model requires no extra testing.

Risk stratification in HFpEF remains challenging. HFpEF is clinically heterogeneous, and patients often differ substantially in comorbidity burden, inflammatory status, haemodynamic profile, and post-discharge trajectory (4, 5). Existing prognostic models have improved understanding of outcome heterogeneity in HFpEF, but their predictor composition, endpoint definitions, and clinical usability vary considerably (5). Some models focus on mortality, whereas others combine hospitalization and mortality outcomes; some incorporate biomarkers or imaging parameters that are not always readily available in routine care. Against this background, a model based on a small number of routinely accessible variables may still be clinically useful, particularly in settings where rapid discharge risk assessment is needed.

The present model is also consistent with the evolving view that HFpEF prognosis is shaped not only by cardiac stress and renal reserve but also by broader systemic vulnerability. In recent years, increasing attention has been paid to inflammatory activation, endothelial dysfunction, metabolic disturbance, and multiorgan interaction in HFpEF (6, 7). This shift is important because rehospitalisation after discharge is often not driven by a single mechanism. Instead, it may reflect the cumulative impact of congestion, impaired reserve, comorbidity burden, vascular dysfunction, and susceptibility to non-cardiac deterioration. The retained predictors in our model fit this broader framework. LnNT-proBNP, GFR, and hypertension represent haemodynamic burden, cardiorenal vulnerability, and comorbidity load, whereas LnSII and LnLp(a) may provide complementary information on inflammatory and vascular risk (10–15).

Within this context, the inclusion of SII deserves particular comment. SII is derived from platelet, neutrophil, and lymphocyte counts and therefore reflects a combination of inflammatory activation, immune imbalance, and thrombo-inflammatory interaction (8). Compared with more specialized biomarkers, it has the practical advantage of being inexpensive, widely available, and easily calculated from routine blood tests. Recent studies have linked higher SII to adverse outcomes in HFpEF and in other cardiovascular populations (8, 16–19). At the same time, SII should not be interpreted as a disease-specific marker for HFpEF. It is influenced by acute infection, chronic inflammatory states, and generalized physiological stress. For that reason, its prognostic value in the present study may lie less in identifying a unique HFpEF mechanism and more in capturing a broader vulnerability state that increases the likelihood of post-discharge rehospitalisation. This interpretation is also compatible with the fact that our endpoint was all-cause rehospitalisation rather than HF-specific recurrence.

The same consideration helps explain why a rehospitalisation model in HFpEF may differ from models designed for mortality alone. Rehospitalisation is a clinically important but heterogeneous endpoint. It may result from worsening congestion, renal dysfunction, arrhythmia, infection, or decompensation of non-cardiac comorbidities. A predictor such as SII may therefore be especially relevant in this setting, because it can reflect a patient's overall inflammatory and physiological burden rather than only the severity of cardiac dysfunction. In that sense, the present findings add to the emerging literature suggesting that inflammatory markers may complement conventional cardiac and renal indices in HFpEF risk assessment (8, 9, 20, 21).

LnLp(a) was also retained in the final model. This finding is of interest because Lp(a) has pro-atherothrombotic and pro-inflammatory properties and may capture vascular risk not fully represented by natriuretic peptides or renal function (10). In HFpEF, where vascular stiffness, endothelial dysfunction, and multimorbidity are common, such information may be relevant. Even so, both LnLp(a) and LnSII should be interpreted cautiously as risk markers rather than causal determinants. Their role in the present model is to improve prognostic stratification, not to establish mechanism on their own.

The overall event rate in this cohort was 31.73%, which should be interpreted in the context of the study design. The endpoint was defined as the occurrence of at least one all-cause rehospitalisation within 1 year after discharge, and therefore captured a broad range of post-discharge clinical deterioration rather than HF-specific recurrence alone. In addition, this was a single-center cohort of hospitalized patients with HFpEF, and local admission practice, comorbidity burden, and follow-up patterns may have influenced the observed event rate. These factors should be considered when judging the generalisability of the model (4, 5).

This study has limitations. This was a single-center study with a modest sample size, and external validation is still required before broader application. Although the validation results were acceptable, the internal validation strategy primarily relied on a stratified 7:3 split of the same dataset. In relatively small samples, this approach may yield less stable estimates than bootstrap-based validation or repeated cross-validation. The lower sensitivity in the validation cohort than in the training cohort may reflect a degree of model instability related to the modest sample size and the split-sample validation strategy. Baseline numeric variables with missing values were handled by median imputation, which may have reduced variability and introduced some degree of bias. All predictors were assessed at baseline only, and changes in inflammatory status, natriuretic peptide levels, and renal function during follow-up were not captured. Finally, because the model was developed for all-cause rehospitalisation, the identified predictors should not be interpreted as specific determinants of HF-related rehospitalisation.

In conclusion, this study developed a clinically accessible model for predicting 1-year all-cause rehospitalisation after discharge in patients with HFpEF. The model integrates inflammatory, haemodynamic, renal, vascular, and comorbidity-related information and showed good discrimination, acceptable calibration, and potential clinical utility in internal validation. Further external validation in broader populations is needed before routine implementation.

## Data Availability

The raw data supporting the conclusions of this article will be made available by the authors, without undue reservation.
